# The association of motivation and perceived social norms with eating behaviors in emerging adults

**DOI:** 10.1080/21642850.2021.2016410

**Published:** 2022-01-02

**Authors:** Namrata Sanjeevi, Jamil M. Lane, Leah M. Lipsky, Denise Haynie, Tonja R. Nansel

**Affiliations:** a Social and Behavioral Sciences Branch, Division of Intramural Population Health Research, Eunice Kennedy Shriver National Institute of Child Health and Human Development, Bethesda, MD, USA; b Department of Counseling and Human Development, University of Rochester, Rochester, NY, USA

**Keywords:** Eating behaviors, motivation, perceived social norms, emerging adults, self-determination theory

## Abstract

**Objective:**

This study examines the associations of eating-related motivation, perceived norms, and their interaction with eating behaviors in emerging adults.

**Design:**

Data are from the NEXT Generation Health Study, a nationally representative sample of US emerging adults. Binominal logistic regression analyses estimated associations of eating behaviors with self-determined motivation, non-self-determined motivation, and perceived social norms. Multiplicative interaction terms between each motivation construct and perceived social norms were tested in the models.

**Results:**

Self-determined motivation was positively associated with intake frequency of whole grains, low-fat dairy, and fruit and vegetables. Soda intake frequency was inversely associated with greater non-self-determined motivation, but not self-determined motivation or perceived social norms. Perceived social norms were positively associated with the intake of whole grains, low-fat dairy, and fruit and vegetables. Perceived social norms did not moderate the association of motivation constructs with eating behaviors.

**Conclusion:**

Self-determined motivation and perceived social norms may be considered in intervention targeting healthful eating behaviors in emerging adults.

## Introduction

During emerging adulthood (approximately ages 18–25 years), developmental transitions may include separation from parental guidance and attaining greater autonomy over life decisions (Arnett, 2000), potentially leading to changes in eating behaviors (Ferrara, 2009). Optimal nutritional intake during this period may promote long-term health habits (Todd, Street, Ziviani, Byrne, & Hill, 2015) and lower the risk of obesity and related comorbidities (e.g. diabetes, hypertension, and heart disease; Nelson, Story, Larson, Neumark-Sztainer, & Lytle, 2008). However, emerging adults generally show poor adherence to dietary guidelines (Banfield, Liu, Davis, Chang, & Frazier-Wood, 2016; Lipsky et al., 2017). Identifying influences on emerging adults’ eating behaviors is thus needed to inform future interventions.

Self-determination theory (SDT; Deci & Ryan, 1985) delineates three distinct types of motivation – intrinsic motivation, extrinsic motivation, and amotivation – each of which may influence initiation and maintenance of health behaviors (McSpadden et al., 2016; Pelletier & Dion, 2007). SDT posits that healthful eating is more likely to occur when it is motivated by one’s desires or values (i.e. intrinsic; Teixeira, Patrick, & Mata, 2011), in contrast to being motivated by external sources such as pleasing others or avoiding guilt (i.e. extrinsic; Deci & Ryan, 2008). Intrinsic motivation for healthful eating is associated with higher fruit and vegetable intake and reduced fat intake (Shaikh, Vinokur, Yaroch, Williams, & Resnicow, 2011; Smit et al., 2018), whereas extrinsic motivation and amotivation are associated with lower fruit and vegetable intake (McSpadden et al., 2016). The basic needs and intrinsic motivation sub-theory of SDT suggests that the basic need for relatedness (social connection) motivates action (Deci & Ryan, 2014). However, little is known about how the effect of motivation on behavior may vary by social context.

Since adolescents and emerging adults adhere to peer social norms to develop peer relationships and maintain connectedness, social norms may motivate behaviors directly through peer modeling or social sanctions (Baker, Little, & Brownell, 2003; Bergquist & Nilsson, 2019; Cialdini & Trost, 1998). Previous studies have shown that perceived norms are positively associated with healthful eating behaviors among adolescents (Pedersen, Grønhøj, & Thøgersen, 2015) and adults 18–46 years of age (Ball, Jeffery, Abbott, McNaughton, & Crawford, 2010), and with greater intentions to maintain a healthy diet (Yun & Silk, 2011). Considering the central role of peer influences during this developmental period, motivation for healthful eating may have a greater impact on behavior when it is reinforced by supportive peer norms. Findings from previous research suggest that perceived social norms may strengthen the association of motives for risk behaviors, such as smoking (Lazuras, 2014) and alcohol use (Choi, Park, & Noh, 2016; Halim, Hasking, & Allen, 2012), with related outcomes. Further, one study has indicated that peer support attenuates the adverse impact of individual-level risk factors, such as low self-esteem, on health-engaging behaviors (Turbin et al., 2006). However, no study has investigated whether social and peer expectations for eating behaviors may strengthen or weaken the influence of individuals’ motivation to eat healthfully. Understanding the interaction of norms and motivation on eating behaviors could inform nutrition interventions that promote healthy eating in emerging adults. Therefore, the objective of this study was to examine the association of diet-related motivation and perceived norms with eating behaviors and determine whether perceived norms moderate the relationship between motivation and eating behaviors in emerging adults. We hypothesize that for emerging adults, motivations and perceived social norms are positively associated with healthful eating behaviors, and stronger health-promoting social norms strengthen the positive relationship between motivation and healthful eating behaviors.

## Methods

### Participants

Data come from wave 4 (1-year after high school; *N* = 2177) of the NEXT Generation Health Study, a nationally representative sample of adolescents in the United States assessed annually. Primary sampling units (i.e. school districts) were stratified by the nine census divisions (Li, Simons-Morton, Brooks-Russell, Ehsani, & Hingson, 2014). Within each census division, school districts were first selected with probability proportional to the total enrollment. A total of 81 schools (response rate = 64%) out of 137 schools agreed to participate during the 2009–2010 academic year. Parental consent and participant assent were obtained from 2,785 participants at baseline, and participant consent was obtained once turning 18 years of age.

## Ethics statement

The study was approved by the Institutional Review Board of the *Eunice Kennedy Shriver* National Institute of Child Health and Human Development.

## Measures

***Self-determined (intrinsic) and non-self-determined (extrinsic) motivation*.** Self-determined and non-self-determined motivations for eating behaviors were measured with scales developed for this study. The self-determined motivation scale consisted of three items: (1) ‘I enjoy it’; (2) ‘It fits with how I see myself’; and (3) ‘It is personally important to me’. The non-self-determined motivation scale included items: (1) ‘I am required to do it’; (2) ‘My parents, other family members, or friends tell me to do it’; and (3) ‘I feel guilty if I do otherwise.’ Response options ranged from 1 = not at all true to 7 = very true. Higher scores reflect higher self-determined or non-self-determined motivation.

***Perceived social norms*.** Perceived peer norms regarding various health behaviors were assessed with items developed for this study, using a format consistent with the assessment of peer norms across a variety of behaviors in prior studies (Hartos, Eitel, Haynie, & Simons-Morton, 2000; Simons-Morton et al., 2016). One item asked participants how important it was to their close friends that they eat a healthful diet (including fruits and vegetables, and limiting junk food, sweets, and fatty foods). Response options on a 7-point Likert scale ranged from 1 = not at all to 7 = extremely. Other scales utilizing somewhat comparable items for measuring peer norms have predicted food group intake (Robinson, Otten, & Hermans, 2016) and demonstrated acceptable test-retest reliability (Pelletier, Graham, & Laska, 2014).

***Eating behaviors*.** Participants reported food group intake frequency using items modified from the Youth Risk Behavior Surveillance System (Youth Risk Behavior Surveillance System, 2014) and the multi-national Health Behavior in School-aged Children study (Vereecken, De Henauw, & Maes, 2005). Respondents were asked, ‘During the past 7 days, how many times did you eat or drink … ?’ Responses ranged from never to four or more times per day. Intake frequency of whole grains, processed meats, low-fat dairy, sweet/salty snacks, and soda were assessed using one item for each group. Fruit and vegetable intake frequency was calculated by summing responses to fruit, 100% fruit juice, green and orange vegetables, and beans (Lipsky et al., 2015). Based on the non-normal distributions and guidelines for healthy eating patterns (U.S. Department of Agriculture and U.S. Department of Health and Human Services, 2020), food group intake frequencies (except fruit and vegetables) were dichotomized as ≥1/day versus <1/day; intake frequency of fruits and vegetables was dichotomized according to the recommended intakes of ≥5/day versus <5/day (Sattar & Forouhi, 2021).

***Demographic characteristics*.** Participants self-reported demographic characteristics including race/ethnicity, gender, and age. Parents reported their educational attainment during the consent process. Family socioeconomic status was estimated using the Family Affluence Scale (Currie et al., 2008), an ordinal measure calculated from participant-reported items including family vehicle and personal computer ownership, frequency of family vacations, and bedroom sharing. The scale ranges from 0 (low affluence) to 7 (high affluence).

## Statistical analysis

All statistical analyses accounted for the complex survey design and were conducted using STATA v. 14 (StataCorp, 2015). Descriptive statistics (means, standard deviations, frequencies, and percentages) were calculated for all variables. Differences in motivation and social norm scores by food group intake frequencies were examined using t-tests. Binominal logistic regression analyses estimated associations of dichotomous eating behaviors with self-determined motivation, non-self-determined motivation, and perceived social norms, entering all independent variables simultaneously and adjusting for gender, race/ethnicity, parent education, and family affluence; reference categories were male for gender; white, non-Hispanic for race/ethnicity; high school or less for parent education; and family affluence was used as a continuous variable. To examine whether perceived social norms moderated the relationship of motivation with eating behaviors, multiplicative interaction terms between each motivation construct and perceived social norms were tested in the models.

## Results

Participants were 19 years old on average in wave 4 (Table 1). Approximately 40% of the sample was male, and 40% were of non-white race/ethnicity. Parent education was well distributed across the three levels (32.2% with high school diploma or less, 39.1% with some college, and 28.7% with bachelor’s degree or higher). Slightly more than 1/5 of the participants consumed fruits and vegetables ≥5 times per day, whereas more than 1/4 of the participants consumed processed meats, snacks and soda more than once per day.

**Table 1.** Sample demographic characteristics (*N* = 2171).

	Mean ± SD		*N*	%
**Age**	19.2 ± 0.5			
**Gender**				
Male			913	41.1
Female			1264	58.9
**Race/ethnicity**				
White, non-Hispanic			862	61.8
Hispanic			557	13.5
Black-non-Hispanic			643	19.8
Other			109	4.9
**Parent education**				
High school or less			748	32.2
Some college			726	39.1
Bachelor’s degree or more			517	28.7
**Family affluence scale**	5.5 ± 1.4			
**Fruit and vegetable intake**^a^				
<5			1680	78.7
≥5			478	21.3
**Whole grain intake**^a^				
<1			1361	59.1
≥1			794	40.9
**Low-fat dairy intake**^a^				
<1			1390	61.7
≥1			766	38.3
**Processed meat intake**^a^				
<1			1561	73.1
≥1			592	26.9
**Snack intake**^a^				
<1			1496	70.3
≥1			657	29.7
**Soda intake**^a^				
<1			1618	72.0
≥1			540	28.0

^a^ Represented as times per day.

Motivation and norm scores were significantly different between fruit and vegetable intake frequencies, such that scores were higher for those with intakes that were ≥5 times per day (Table 2). Self-determined motivation and norm scores also were significantly higher for adolescents whose whole grain and low-fat dairy intake frequencies were ≥1 times per day. Non-self-determined and self-determined motivation scores were significantly lower for adolescents whose soda intake frequencies were ≥1 times per day. Other differences in scores between the food group intake categories were not statistically significant.

**Table 2.** Differences in motivation and social norm scores^a^ by food group intake frequencies.

	Intake frequency (times per day)											
	Fruits and vegetables		Whole grains		Low-fat dairy		Processed meats		Snacks		Soda	
	<5	≥5	<1	≥1	<1	≥1	<1	≥1	<1	≥1	<1	≥1
**Motivation type**												
Self-determined	11.2 ± 4.9	13.1 ± 5.0***	11.1 ± 5.1	12.4 ± 4.6***	11.1 ± 5.0	12.5 ± 4.7***	11.4 ± 4.9	12.2 ± 5.0	11.5 ± 4.9	11.8 ± 5.0	11.8 ± 5.1	11.1 ± 4.5*
Non-self-determined	6.0 ± 3.7	7.8 ± 5.0***	6.3 ± 4.1	6.5 ± 3.9	6.1 ± 4.0	6.9 ± 4.1*	6.3 ± 4.0	6.8 ± 4.3	6.4 ± 4.1	6.4 ± 4.0	6.7 ± 4.3	5.6 ± 3.4*
**Social norms**	2.8 ± 1.7	3.5 ± 1.8**	2.8 ± 1.8	3.2 ± 1.6*	2.7 ± 1.7	3.3 ± 1.7*	2.9 ± 1.8	3.0 ± 1.7	2.9 ± 1.7	3.0 ± 1.8	2.9 ± 1.8	3.0 ± 1.6

*p < 0.05, **p < 0.01, ***p < 0.001.

^a^ Represented as mean ± SD.

In models including both motivation types and perceived norms (Table 3), greater self-determined motivation was associated with greater odds of consuming whole grains and low-fat dairy ≥1 times per day. Self-determined motivation also was related to increased odds of consuming fruits and vegetables 5 or more times per day, but was not associated with the processed meat, soda or snack intakes. Greater non-self-determined motivation was associated with greater odds of consuming fruits and vegetables 5 or more times per day, but lower odds of consuming soda ≥1 times per day. Non-self-determined motivation was not associated with intake frequencies of all other food groups. Perceived social norms was associated with higher odds of eating whole grains and low-fat dairy ≥1 times per day, but not with odds of consuming soda, snacks and processed meat. Interaction terms between motivation types and perceived social norms on intake frequency of all food groups were not statistically significant.

**Table 3.** Adjusted odds ratio^a^ relating motivation and social norm scores to food group intake frequency^b^.

Independent variables	Food group intake frequency (times/day)					
	Fruit and vegetables	Whole grains	Low-fat dairy	Processed meats	Snacks	Soda
**Motivation type**						
Self-determined	1.04 (1.002, 1.08) *p* = 0.04	1.06 (1.03, 1.09) *p* < 0.001	1.05 (1.01, 1.08) *p* = 0.01	1.02 (0.98, 1.07) *p* = 0.33	1.02 (0.97, 1.08) *p* = 0.28	0.98 (0.95, 1.01) *p* = 0.25
Non-self-determined	1.07 (1.03, 1.12) *p* = 0.004	0.98 (0.93, 1.02) *p* = 0.24	1.01 (0.95, 1.07) *p* = 0.70	1.03 (0.98, 1.07) *p* = 0.23	1.00 (0.94, 1.06) *p* = 0.40	0.93 (0.88, 0.98) *p* = 0.03
**Social norms**	1.13 (0.99, 1.28) *p* = 0.07	1.11 (1.01, 1.24) *p* = 0.04	1.16 (1.01, 1.33) *p* = 0.03	0.98 (0.89, 1.08) *p* = 0.71	1.01 (0.94, 1.08) *p* = 0.79	1.03 (0.95, 1.11) *p* = 0.43

^a^ Estimated from logistic regression entering all independent variables simultaneously and adjusted for sex, race/ethnicity, parent education, and family affluence score, and accounting for the complex survey design.

^b^ Reference categories were <5 times/day for fruits and vegetables and <1 time/day for all other food groups.

## Discussion

This study examined associations of motivation for healthful eating (i.e. self-determined and non-self-determined) and perceived social norms with eating behaviors among emerging adults and investigated whether social norms moderate the relationship of motivation with eating behaviors. In models including both motivation types and social norms, self-determined motivation was positively associated with intake frequency of healthful foods, while only the associations of non-self-determined motivation with lower soda and greater fruit and vegetable intake frequencies were statistically significant. Perceived social norms was positively associated with odds of intake of healthful food groups. The modest effect sizes observed for these associations suggest that additional individual, environmental and social factors could impact eating behaviors. Findings did not support our hypothesis that perceived social norms would moderate the association, suggesting that health-promoting perceived norms do not strengthen the association of motivation constructs with healthy eating behaviors.

Positive associations of self-determined motivation with eating behaviors observed in this study are consistent with research indicating that greater self-determined motivation is associated with more healthful eating behaviors in emerging adults (McSpadden et al., 2016), higher diet quality in young adult women (Pelletier & Dion, 2007) and greater dietary adherence in adolescents with type 1 diabetes (Austin, Senécal, Guay, & Nouwen, 2011). Further, non-self-determined motivation was related to lower soda and greater fruit and vegetable intake frequencies in the current study. This finding differs from previous research demonstrating that non-self-determined motivation is not associated with healthful eating behaviors (Trudeau, Kristal, Li, & Patterson, 1998) and may facilitate unhealthy eating behaviors (Leong, Madden, Gray, & Horwath, 2012) in older adults. Given that individuals’ motivational strategies may evolve over time (Heckhausen, Wrosch, & Schulz, 2010), discrepancy in findings may be attributed to differences in the age of the study samples. Nevertheless, findings from this and previous studies suggest that motivation for healthful eating may play a role in shaping eating behaviors of individuals.

Additionally, the associations of perceived norms with intake frequency of healthful food groups observed in this study are consistent with previous findings that stronger perceived norms are associated with greater intention to consume fruit and vegetables (Smith-McLallen & Fishbein, 2008; Yun & Silk, 2011). Consistent with theoretical perspectives suggesting that perceived social norms may influence motivation (Cialdini & Trost, 1998; Cialdini, Reno, & Kallgren, 1990), modest associations of social norms with both motivation types were observed in this study; however, there was no evidence that social norms moderate the association of motivation with eating behaviors.

Several limitations should be noted. This is a cross-sectional, observational study; therefore, causality cannot be inferred. Self-report data is susceptible to response bias, although there is no known objective measurement of social norms or motivation. Although food group intake assessed via dietary screener may be more prone to measurement error, the screener is considered adequate for measuring population-level eating behaviors (Lipsky et al., 2015). The use of a single-item to measure peer norms for eating behaviors could have biased findings toward null; however, multi-item scales used in previous studies assess peer norms related to several health behaviors, such as diet, exercise and screen time, with a single item for each health behavior (Rice & Klein, 2019; Turbin et al., 2006). The study findings are strengthened by the large, diverse, nationally representative sample of US adolescents. Further, accounting for hypothesized covariates supports the internal validity of these results. To our knowledge, this is the first study to investigate the role of perceived norms as a moderator of the association between motivation constructs and eating behaviors among emerging adults.

## Conclusion

The current study expands the literature on how motivation and social norms influence eating behaviors. In this nationally representative sample of emerging adults, self-determined motivation and perceived social norms were associated with more frequent intake of healthful food groups; however, non-self-determined motivation was not consistently associated with healthful eating behaviors. The absence of a moderating effect of social norms suggests that self-determined motivation was positively associated with healthy eating behaviors regardless of the strength of social norms. Findings suggest that interventions incorporating methods to increase self-determined motivation and positive social norms could support healthful eating behaviors in emerging adults. The modest strength of the observed associations suggest that substantial increases in motivation and social norms may be required to achieve improvements in eating behaviors, and therefore interventions targeting multiple influences are likely required.

## Data Availability

Data used for this analysis will be made available upon request. The design and analysis plans were not preregistered.
